# Instance Segmentation to Estimate Consumption of Corn Ears by Wild Animals for GMO Preference Tests

**DOI:** 10.3389/frai.2020.593622

**Published:** 2021-01-29

**Authors:** Shrinidhi Adke, Karl Haro von Mogel, Yu Jiang, Changying Li

**Affiliations:** ^1^ Institute of Artificial Intelligence, University of Georgia, Athens, GA, United States; ^2^ Bio-Sensing and Instrumentation Laboratory, College of Engineering, University of Georgia, Athens, GA, United States; ^3^ Biology Fortified, Inc., Middleton, WI, United States; ^4^ Horticulture Section, School of Integrative Plant Science, Cornell AgriTech, Cornell University, Geneva, NY, United States; ^5^ Phenomics and Plant Robotics Center, University of Georgia, Athens, GA, United States

**Keywords:** deep learning, mask R-CNN, instance segmentation, GMO, image processing

## Abstract

The Genetically Modified (GMO) Corn Experiment was performed to test the hypothesis that wild animals prefer Non-GMO corn and avoid eating GMO corn, which resulted in the collection of complex image data of consumed corn ears. This study develops a deep learning-based image processing pipeline that aims to estimate the consumption of corn by identifying corn and its bare cob from these images, which will aid in testing the hypothesis in the GMO Corn Experiment. Ablation uses mask regional convolutional neural network (Mask R-CNN) for instance segmentation. Based on image data annotation, two approaches for segmentation were discussed: identifying whole corn ears and bare cob parts with and without corn kernels. The Mask R-CNN model was trained for both approaches and segmentation results were compared. Out of the two, the latter approach, i.e., without the kernel, was chosen to estimate the corn consumption because of its superior segmentation performance and estimation accuracy. Ablation experiments were performed with the latter approach to obtain the best model with the available data. The estimation results of these models were included and compared with manually labeled test data with *R* ^2^ = 0.99 which showed that use of the Mask R-CNN model to estimate corn consumption provides highly accurate results, thus, allowing it to be used further on all collected data and help test the hypothesis of the GMO Corn Experiment. These approaches may also be applied to other plant phenotyping tasks (e.g., yield estimation and plant stress quantification) that require instance segmentation.

## 1 Introduction

Corn is one of the world’s most important crops and is produced both traditionally and with genetically modified organisms (GMO) (FAOSTAT, 2018). To obtain certain agriculturally desirable traits, such as resistance to pests, herbicide tolerance, and drought tolerance, specific corn varieties have been genetically engineered. Despite research on its safety and equivalence to traditional varieties, questions continue to be raised by members of the public regarding its safety and edibility. Since its introduction, there have been mixed views on GMO foods and crops and GMO corn is no exception for this. An early study summarizes the environmental benefits and risks of GMO corn (Gewin, 2003). In 2008, a United States (US) grower observed that mice preferred non-GMO corn over GMO corn (Roseboro, 2008). To test this, another grower repeated the experiment and published his results online stating that “The squirrel could have switched to GMO, but it did not. It knew it was different” (Roseboro, 2013). Another study done by an Italian group claimed GMO corn to be toxic (Séralini et al., 2012). This led to several studies and debates amongst research community and the corn industry (Butler, 2012). The initial study was retracted because of a lack of detailed analysis and insufficient evidences, but this did not stop further studies and debates on GMO corn. Further studies in favor and against GMO corn were done which is collectively reviewed by Chassy and Tribe (2010) and some of them clarified that “*animals are not biased to organic corn.*”

During this time, the hypothesis was formulated that wild animals, specifically squirrels and deer, can sense differences between GMO and non-GMO corn and avoid GMO corn or prefer non-GMO corn when given a choice between the two. To test this hypothesis, a nation-wide community science project called “The GMO Corn Experiment” was started in 2015 that gathered the image data of GMO and non-CMO corn set outside by volunteers in their yards (Haro von Mogel and Bodnar, 2015). The next challenge of this experiment was to estimate the precise consumption of all the GMO and non-GMO corn samples based on the complex image data obtained to address the hypotheses being tested in the study. In the experiment, there were two choices of corn for animals kept side-by-side with bar-coded labels to keep volunteer community scientists blinded to the identity of the ears. On a larger scale, this study can serve as a model for studying animal preferences of GMO vs. non-GMO food in a publicly accessible manner. The experiment recruited volunteers ranging from families with children, to school classrooms, and adults, which necessitated striking a balance between simplicity and thoroughness in the data collection strategy. Volunteers were asked to take images of one side of the ears of corn before and after 24 h, but had the option to add additional data and observations. This helped provide consistent samples of each ear of corn, however, the image data still presented challenges to analyze. Visual observations of the ears could be unreliable, as well as time-consuming and tedious to accurately estimate the consumption of each ear used in the experiment due to the large number of images and the time required for each image. Multiple observers would be needed to overcome individual biases, and any error could result in supporting a false hypothesis and affect future research. Finally, the wide range of image orientations, dimensions, quality, and lighting conditions would make traditional computer analyses difficult to perform, so a more robust method of analyzing community science-generated images was needed.

Computer vision can play an important role in estimating the consumption rate automatically. One of the challenges is to identify corn ears from the images and distinguish between the consumed and the non-consumed parts of the corn. Thus, a detection algorithm is needed that will distinguish between different parts of the corn, and based on its detection, compute the consumption percentage. Object detection and instance segmentation are two common categories in computer vision used to detect, classify, and segment images based on predefined/labeled classes. In object detection, the object’s location in the given image is identified, whereas instance segmentation detects and delineates each distinct object of interest with the corresponding pixels in the image. There have been many studies on segmentation of plants/crops to detect different diseases and various image processing techniques have been reviewed by Hamuda et al. (2016). For instance, one study used color transformation from the RGB to CIELAB color space to segment blight in corn leaves (Sukmana and Rahmanti, 2017) while another study used color features and K-means clustering to segment and identify crop diseases (Kumar and Jayasankar, 2019). Compared to traditional image processing, deep learning has shown promising results in fruit detection, plant phenotyping, and yield estimation tasks (Koirala et al., 2019; Jiang and Li, 2020). For example, one study proposed a method to augment training images using synthetic data to train a deep learning model Mask R-CNN to segment individual leaves of a plant (Ward et al., 2018).

Image instance segmentation was best suited to estimate the consumption of corn because it is crucial to know the exact area of the individual ear of corn and the consumed part of the corn. The problem is with the subset of instance segmentation, in which there may be more than one label for single pixel, a phenomenon known as multi-label segmentation. This poses challenges as most current algorithms cannot handle this type of task. Recent advances in Convolutional Neural Networks led to a variety of frameworks that can be used to perform instance segmentation on different levels (Hafiz and Bhat, 2020). One of the most successful and popular approach was the Mask R-CNN framework which efficiently detects the object while simultaneously generating a high quality segmentation mask for each instance (He et al., 2017). It achieved an average precision (AP) of 37.1% with a speed of five frames per second on benchmark datasets. Moreover, Mask R-CNN has proven efficient in segmenting leaves (Ward et al., 2018) and nuclei (Johnson, 2018) which relied upon a limited amount of data for training. In this paper, an automated algorithm is proposed that uses the masks produced by the Mask R-CNN method to estimate the area of the consumed part of the corn as well as the entire corn, which results in a percentage consumption/eaten. Specific objectives of this study were to:

1 Compare the data labeling approaches that were used for model training.
2 Perform ablation experiments for model training parameters and identify an optimal training data size.
3 Evaluate the model performance in segmentation using manually labeled ground truth.

## 2 Materials and Methods

**FIGURE 1:**
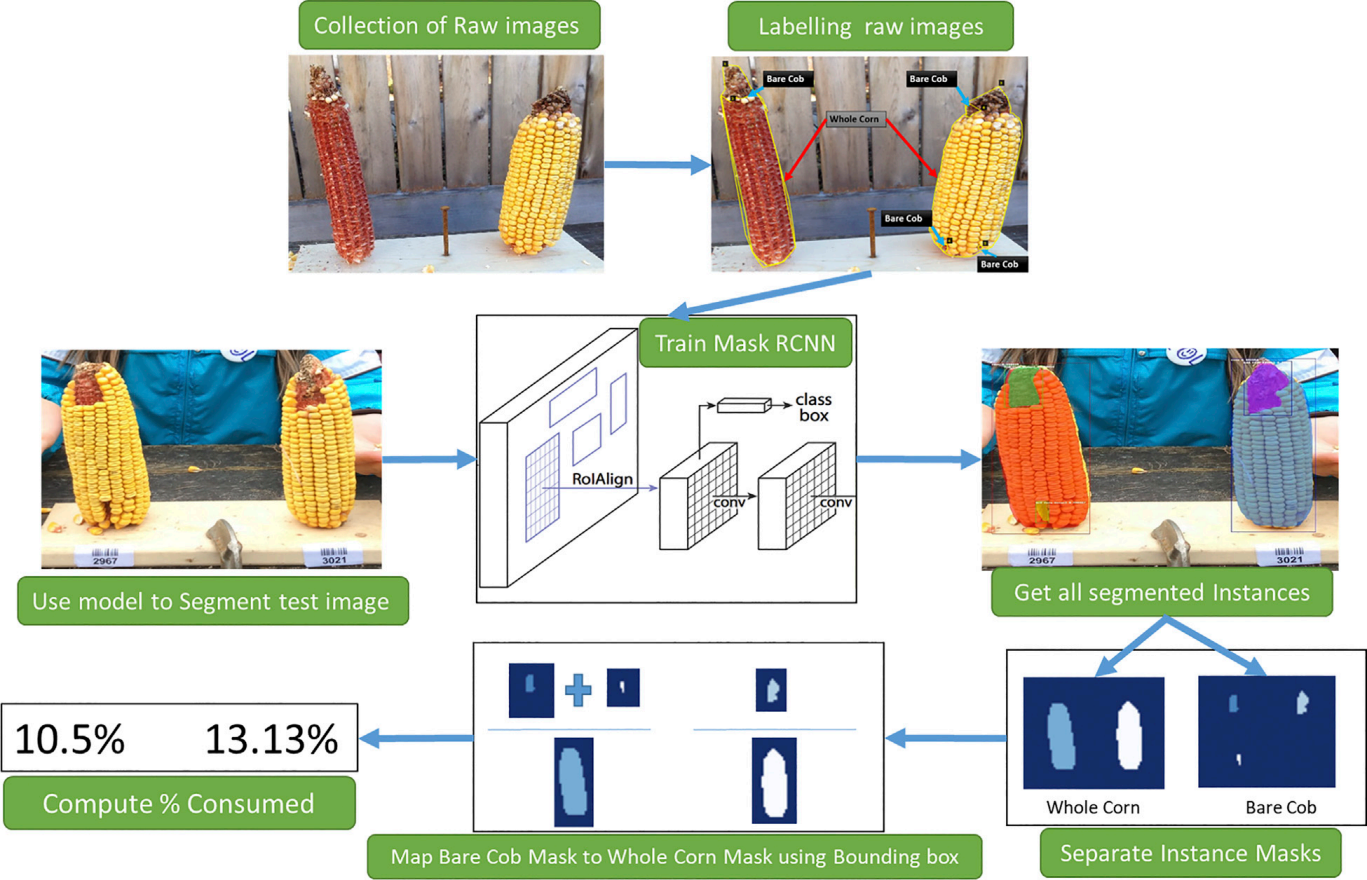
An overview of stages of consumption estimation from raw images. Collected raw images are labeled manually and used to train the Mask R-CNN model. The instances segmented by the model and their masks are then processed to estimate the consumption.

The proposed workflow is presented in Figure 1 to estimate corn consumption from raw images collected. This involves three primary tasks: 1) Raw data labeling, 2) Training Mask R-CNN to segment corn instances, 3) Consumption estimation from segmentation results.

### 2.1 Image Collection and Annotation

#### 2.1.1 Data Source and Pre-screening

Experiment kits were distributed to volunteer community (non-academic) scientists in the United States containing two experiments, each consisting of a pair of size-matched GMO and non-GMO corn, a feeding stand, and instructions for conducting the experiments. They placed experimental setups in their backyard or some kind of open space, thereby offering one GMO and one non-GMO corn to wild animals in the same environmental conditions, taking observations at 24 h intervals. Each ear of corn was labeled with an unique bar code without cultivar information to avoid potential human bias. A total of 630 images were provided at the start of the project, which needed to be analyzed in a reliable and repeatable manner. Based on visual observations, pairs of before/after images from each experiment were pre-screened, and if both of the ears of corn in the experiment were consumed between 0–5%, 5–95% or 95–100%, that image was placed in its respective category. In the case of mixed consumption, they were labeled as 5–95%. This approach allowed for the consideration of the potential variations in consumption for training the segmentation model. Table 1 shows the category-wise statistics of the images considered for further annotation stage.

**TABLE 1:** Image categorization after pre-screening.

**Category**	**Total images**	**Selected for annotation**	**Selected for training**
0–5%	210	143	95
5–95%	370	257	175
95–100%	50	50	30
Total	630	450	300

Multiple images were gathered from a single kit over the course of 24 h intervals, (e.g. the same corn ear would be consumed more on the second day than on the first day). For training purposes, images of the same ears of corn at different phases of the experiment were considered (initial, intermediate, final consumed image). This allowed more data to be collected with fewer experiment kits. After collecting the raw images in various conditions, a total of 450 images were selected from these categories for manual annotation and labeling. In this dataset, ambiguous images are those in which it could not be identified whether the corn is present or not, and if present, consumption could not be estimated. Based on these considerations, images for training was selected based on following criteria:

- Corn ears present in the image should be identified easily by the human eye.
- The image should be high-resolution and have legible brightness and contrast.
- The skewing of the image, (i.e. rotating the image at various angles, changing the brightness/contrast, applying blur) should not result in ambiguous image.
- The image can contain other objects than corn such as a chair, table, person, toy, etc.

#### 2.1.2 Labeling Approaches

The sorted image data were then labeled using VGG’s Image Annotator (VIA) tool (Dutta et al., 2016). For this study the whole corn in the image as well as the consumed parts of the corn were needed to be identified. To achieve this, the masks were labeled in many different ways. However, predicting exact and adequate segments posed the biggest challenge to the estimates. The output of the model is based on the segments and class masks given for training. To estimate consumption, it is possible to compare the eaten part, i.e., the bare part of corn ear, with the total visible part of the corn ear. Also, an individual corn kernel or cluster of kernels might have been considered as separate classes. Two of many possible approaches that consider two distinct classes were attempted.

**FIGURE 2:**
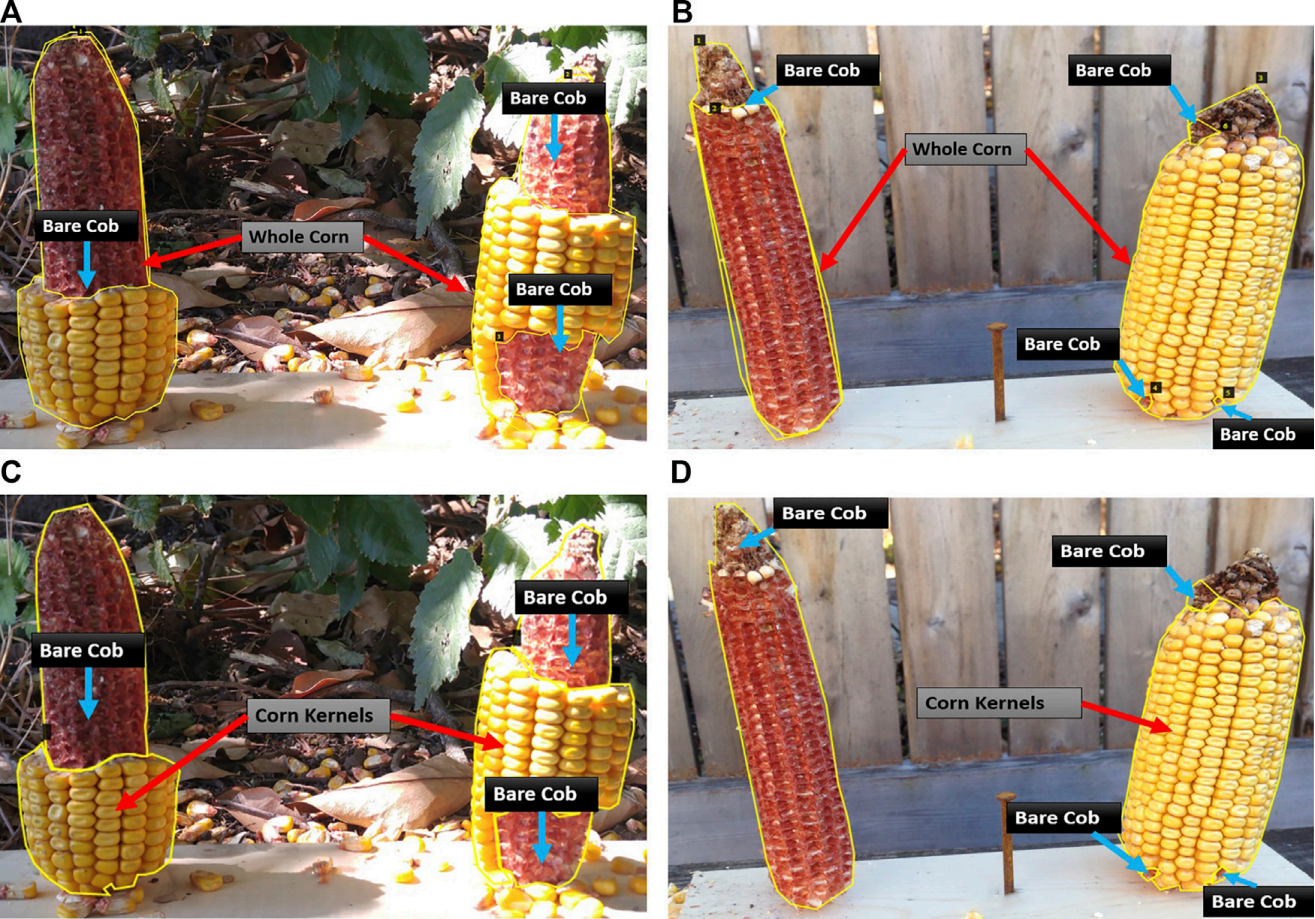
Illustration of two data labeling approaches. Representative images with different levels of consumption and corresponding masks. In Approach 1 **(A–B)**, the visible part of a whole corn ear and distinguishable bare part segments are labeled as two mask classes. In Approach 2 **(C–D)**, intact corn kernels and bare part segments are labeled as two mask classes.

First, the whole corn and bare corn ear were considered as two distinct classes (Approach 1), while in the second approach, the clusters of intact corn kernels along with bare corn ears were considered as two separate classes to segment (Approach 2). The two approaches differ at the image labeling level. For Approach 1, masks were drawn for whole corn ears and bare cob parts were used for consumption estimation, while in Approach 2, masks were drawn for corn kernels and bare cob parts, the sum of which equals a whole visible corn ear (which is not considered as a separate class for segmentation but computed later by adding these two classes). Figure 2 illustrates sample images from the training dataset for both approaches.

After performing the experiments with these approaches, which are discussed in later sections of this paper, manual labeling of all 450 images was continued for Approach 1. Table 2 provides the details of data partitions and the corresponding number of labeled masks for Approach 1. It should be noted that a normal image in the dataset has two corn ears. The table shows that there are certain images present in the dataset, which contains only one corn ear. These images may not be useful for comparing two corn ears, but they were selected to improve image segmentation accuracy and to avoid overfitting the model to specific features in images having two corn ears.

**TABLE 2:** Data partitioning and number of masks for labeling approach 1.

**Labeled dataset**	**# Of images**	**# Of corn masks**	**# Of bare cob masks**
Training	300	593	846
Validation	125	248	408
Testing	25	50	79

### 2.2 Corn Segmentation

#### 2.2.1 Mask R-CNN

As the Mask R-CNN architecture has proven successful in a wide range of applications requiring instance segmentation, it was chosen for the present study. It consists of two stages: the first stage scans the image and generates areas with a high probability of containing an object of interest, often referred to as *proposals*, and the second stage is responsible for the classification of these proposals to generate bounding boxes and *masks* for each detected object. To build the basic network, we used Matterport’s (Abdulla, 2017) implementation of Mask R-CNN and performed ablation experiments with modified configurations.

Based on experiments with this implementation for different segmentation tasks such as deep leaf segmentation (Ward et al., 2018) and nuclei segmentation (Johnson, 2018; Naylor et al., 2018), widely used common parameters were chosen for the training. Also, ResNet-50 was (He et al., 2016) as the backbone network to detect features. To improve standard feature extraction, a feature pyramid network (FPN) (Lin et al., 2017) was added to the network. Preliminary trials with small samples were conducted to tune the hyper-parameters such as learning rate, non-max suppression threshold, and training ROIs per image. The remaining parameters were left unchanged from Matterport’s original implementation. The training followed a predetermined backpropagation schedule with a stochastic gradient descent (SGD) optimizer, L2 Regularization with a weight decay of 0.0001, a learning rate of 0.001, and cross entropy loss functions for various losses in the network.

During the preliminary trials, the model tended to overfit because of a small sample size of 50, as the model learned the features only specific to those training images, such as the number of corn ears present and the position and alignment of corn ears in the picture. Therefore, to prevent model overfitting, image augmentation (Jung, 2018) methods were used to enhance the dataset diversity. These augmentations include random image flips (left/right/up/down) and rotations (90°, 180°, 270°) along with Gaussian blur, color multipliers. The gaussian blur and color multiplier takes care of variations in the dataset such as image focus, distance (pan/zoom) of object from camera, color variations in objects as well as background. Also, to avoid having the images with similar test-kit positions i.e., two vertical ears in the center of the image, we considered the images with skewed ear positions, various image angles and kits having only one corn ear in the training dataset.

The dataset has images of different resolutions ranging from 640 × 480 to 4,080 × 3,072 with an average of 1,280 × 760. For training, the input image size was limited to 1,024 × 1,024 with the help of Matterport’s utility methods that uses the standard bilinear interpolation to resize the image. A batch size of two was used because of the GPU memory limit (NVIDIA GeForce GTX1080Ti) and generally, steps per epoch are decided by batch size along with number of training samples. In this work, training configurations were optimized by observing the differences between training and learning methods and to observe which one can perform better in terms of estimating the corn consumption.

#### 2.2.2 Ablation Experiments

To begin the consumption estimation, a better segmentation model was trained by performing various ablation experiments, which led to the effective end model used for testing. Labeling approach and training sample size were the two primary factors considered in the ablation experiments.

**1) Labeling Approach comparison.** The overall segmentation problem was simplified at the data labeling level. All the raw images were labeled according to one of the two approaches explained in the previous section. Instead of labeling the *entire* dataset twice to arrive at a better labeling approach for further experiments, this test was performed at the beginning with a smaller dataset. For initial comparison purposes, out of the 450 raw images, 70 were selected and manually labeled using both approaches. For this experiment, 50 training images and 20 validation images were used. Identical network configurations were selected for both labeling methods. This experiment indicated which labeling approach to follow for the remaining raw images.

Labeling each image for the two approaches took considerable time. In a standard image with two corn ears, both class instances could be labeled in one image within 3 min on average using Approach 1, while Approach 2 took 4.5 min on average. This was because, in a single image total instances of corn kernels can be more than total whole corn instances. There will be at most two whole corn instances but can be zero or multiple corn kernels present in one image, labeling multiple corn kernel instances contributed to more time in Approach 2. In the end, the Mask R-CNN model trained using images labeled by approach 1 were referred to as *Model one* while the one by approach 2 as *Model 2*.

**2) Training sample size effect.** One of the challenges in training effective deep learning models is the limited amount of training data. The sparsity of labeled images in the agricultural domain is a common problem for segmentation model failures resulting from overfitting to a small sample size. The number of training images required is not fixed, but they are domain and application specific. To ascertain the minimum number of training images for a good segmentation performance, this experiment was performed.

To answer this question, multiple models were trained with a different number of training images with an approach selected from the above comparison. Starting with 50 images, models were trained on increments of 50 images, up to 300 images, while keeping an uniform size in the validation dataset. In preliminary tests, it was observed that, when the selected training samples contained only the images from a certain category, (e.g. “95–100% consumed”), the resultant model performed poorly on the remaining categories, which resulted in inaccurate image segmentation. To address this data imbalance, each training procedure was performed five times by selecting the training images randomly from each category. Thus, a total of 30 different segmentation models were trained with six different sample sizes.

Apart from the major experiments mentioned above, the transfer learning phenomenon applied to this use case was also examined. During preliminary testing, one of the Mask R-CNN model was trained by initializing random weights at the beginning. Then, this model was compared to a model trained on pre-trained weights on the MS-COCO dataset (Lin et al., 2014). For better segmentation of the background from the corn ears in the image, further models were trained using COCO initial weights.

#### 2.2.3 Evaluation Metrics

To evaluate the performance of the above ablation experiments, a comparison was made of the training procedures as well as the results on labeled test dataset. Below are the metrics considered for evaluating these experiments.

**1) Jaccard Index.** It is important to predict the mask accurately as the area to be calculated is based on the mask. This can be verified by standard metrics of Jaccard Index, a.k.a. Intersection over Union (IoU), which is the ratio between the overlap of a predicted mask and the actual ground truth mask and the area of union between the predicted mask and the ground truth mask. Then the weighted mean IoU was computed for all the predicted instances of each class in an image and average all the images in the dataset. In both models, class “*bare cob*” is present, while *Model 1* has “*whole corn*” and *Model 2* has “*corn kernel*” as the second class, respectively. Mean IoU will be a key metric used to evaluate the segmentation performance of these models.

**2) Mean Average Precision.** For instance segmentation, it is important to compute the precision and recall achieved by the model in addition to IoU. Precision is defined as a ratio of true positives over both true and false positives. Recall is defined as the ratio of true positives over both true positives and false negatives. Precision and recall are computed over a range of different IoU thresholds (typically 0.5 to 0.95 in steps of 0.05). The average precision (AP) is the averaged precision for all classes for one input image. The mean of APs is the mean average precision (mAP) over all images.

**3) PR Curve.** Along with precision values, the recall of these models can be visualized better in terms of a precision-recall (PR) Curve. The area under the PR curve for a certain IoU threshold is nothing but the mAP for that model. We can plot the PR curve for different IoU threshold values that are especially close to the model’s mean IoU to ascertain how well it is performing on all of the ground truth instances.

The main criteria for selection of a better labeling approach involves a high mean IoU value, a near ideal PR curve for different IoU thresholds, and thus a high mAP value. Additionally, it is important to consider the complexity in computing the consumption ratio with segmentation masks of two different labeling approaches. The ideal approach should be less complex in terms of detection of instances. It should present a significant overlap of predictions and ground truth masks.

### 2.3 Corn Consumption Estimation and Comparison

#### 2.3.1 Calculation of Corn Consumption Ratio

Once a network was trained to segment the required classes, distinct corn ears in the image would need to be identified to estimate the consumption. For this, a straightforward method was used for preparing a list of all distinct class instances and compute the sum of individual pixels. The detection results of the model provides all instances of a whole corn, the consumed part of the corn, and corn kernels (in Approach 2). Each instance has its mask pixels and the bounding box coordinates. This output was further processed using custom Python scripts to arrive at final consumption estimations.

After segmenting the image into the whole corn and its corresponding parts—bare cob part and corn kernels (as illustrated in Figure 1), the next step is to group the segmentation results to map all parts to respective corn ears. This is done by using the bounding box created by Mask R-CNN’s bounding box detection layer. For example in Approach 1, the individual whole corn instances are first separated and then grouped among all other segmented instances of bare cob parts in their bounding box. This provides mapping of all whole corn instances and their bare cob parts.

Finally, the consumption was determined by calculating the ratio of *total pixels of all individual consumed or bare cob parts of the corn* over *total pixels of that corn* (In Approach 2, the total pixels of corn is the sum of the pixels of all bare cob parts and the pixels of all kernel parts).

#### 2.3.2 Evaluation Methods

During the pre-screening and categorizing stage, the images were manually annotated by authors. To verify the consumption value obtained from manual annotations, five human observers rated the test images. The averages of all observed consumptions were verified with the values obtained from manual annotations. In this comparison, the manual annotations were considered as ground truth for consumption estimation.

The consumption was calculated individually for each segmented corn ear. It should be noted that a variation of ±4% in consumption estimation was observed because of configurations of the test environment. This can be visualized by a scatter plot with a linearly fitted line. We observed failure in segmentation for a few of the models trained on less samples, which led to inaccurate consumption estimations. These cases were treated as outliers while evaluating consumption estimations. The outliers were not considered in fitting the line. The consumption of left and right corn ears was then compared, and the results can be viewed in the confusion matrix compared from human observations.

## 3 Results and Discussion

### 3.1 Labeling Approach Comparison

**FIGURE 3:**
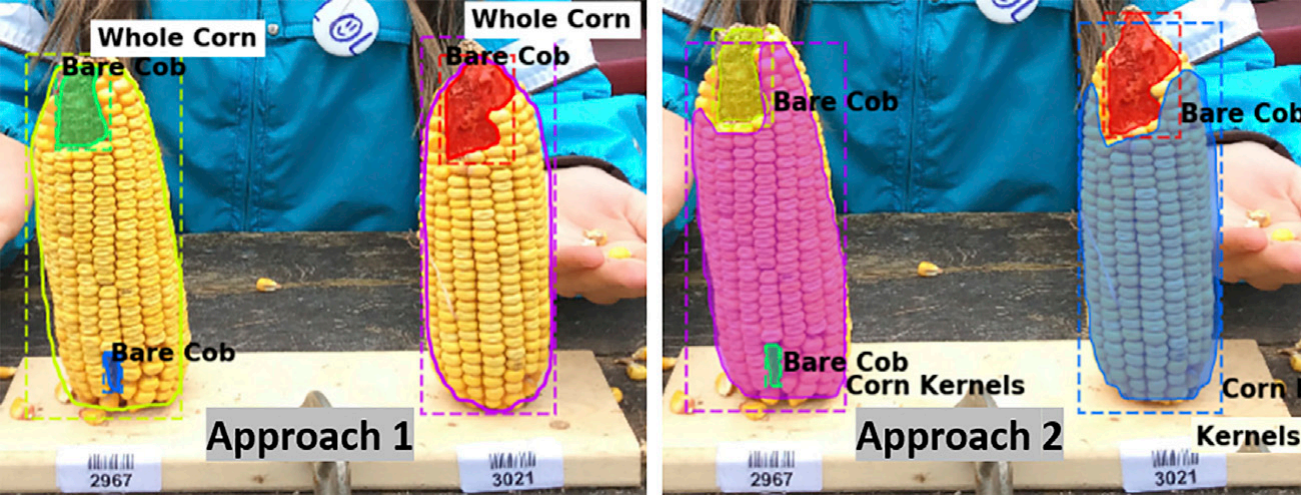
Segmentation using two labeling approaches. For better visualization masks are not shown for the whole corn class in Approach 1 **(left)**, while both class masks are shown in Approach 2 **(right)**.

The metric values for the two annotation approaches were compared and the aim was to select one labeling approach that will be used for further experimentation and labeling the required training data. It can be seen that *Model one* gave better results on the overall test dataset (Table 3). Figure 3 shows example outputs using the two approaches. Approach 2 missed a significant portion of both classes that can be seen by observing the instance masks. In addition, Approach 2 segmented the inner bare part as both classes due to its relatively small size and surrounding kernels, which was a false positive for corn kernel class and can result in an inaccurate estimation of consumption.

**TABLE 3:** Labeling approach comparison.

**Metric**	**Model 1**	**Model 2**
Mean IoU for bare cob	0.61	0.51
Mean IoU for corn kernel	–	0.77
Mean IoU for whole corn	0.87	0.64
mAP	0.57	0.48

Overall, labeling Approach 1 led to a better performance than Approach 2 which can be seen from above results. Compared with Approach 2, Approach 1 increased the segmentation accuracy by approximately 10% and 23% for bare cob and whole corn, respectively, which were substantial improvements for a two-class segmentation problem. This occurred primarily because segmentation of bare cob and whole corn was simpler than that of bare cob and corn kernels. First, a whole corn ear had a relatively predictable conical shape regardless of how much of it is consumed, whereas corn kernel parts could be in any shapes and locations based on the consumption of the ear. Given the same number of training images for a whole corn ear, a Mask R-CNN model could easily learn adequate feature representations, resulting in better segmentation accuracy. Second, Mask R-CNN could not achieve a perfect segmentation of objects with complex boundaries, such as bare cob and corn kernel parts both of which had variable instance mask boundaries that were not as obvious as the conical whole corn. In particular, the boundaries of the two parts were dramatically variable because of natural uncertainties in the experiments such as ear placement height and ear size. Additionally, since it was not possible to predict how the corn ears were consumed by animals and thus the remaining bare parts and kernel parts could vary. Approach 2 included both classes (bare cob and corn kernels) and increased the difficulty of training a model for such a segmentation task. In addition, the two annotation methods had different labeling cost and model training time.

Proceeding with Approach 1, it was determined that the consumption estimation was faster for all the different image types because of the easy computation involved in detecting the whole corn area. Approach 2 required extra computations to identify the appropriate kernels and bare parts for a single corn based on its bounding box. This was not the case with Approach 1, since it gives the bounding box of a whole corn that already has the bare part. Thus for initial experimentation purposes, Approach 1 performed considerably well.

### 3.2 Effect of Sample Size

Various models with different training sample sizes were compared in terms of key performance metrics (Table 4). As more images were added to training, the segmentation improved generally. For bare cob class, the model with a sample size of 150 slightly under performed compared to model with 100 samples and thus lowering the total mAP by 0.014. With two-sided t-tests (Supplementary Table S1–S3), the performance metrics were statistically analyzed to find the significant improvements corresponding to various sample sizes. Sample increment in most of the early sample sizes found to be insignificant with respect to IoU. However, we achieved significant improvement in mAP with all 300 images. Therefore, the use of all 300 training images is beneficial to the best performance in the present study. In the future, annotating 50 to 150 images would initiate a good baseline model at an affordable cost. With active learning methods, additional instances important to model performance improvements could be identified and labeled with minimized human efforts.

**TABLE 4:** Effect of training sample size on segmentation performance (mean ± standard deviation).

**Sample size**	**50**	**100**	**150**	**200**	**250**	**300**
Bare cob IoU	0.606 ± 0.04	0.636 ± 0.02	0.626 ± 0.03	0.661 ± 0.02	0.656 ± 0.04	0.670 ± 0.02
Whole corn IoU	0.868 ± 0.01	0.876 ± 0.01	0.885 ± 0.01	0.886 ± 0.01	0.888 ± 0.01	0.893 ± 0.01
mAP	0.574 ± 0.01	0.592 ± 0.01	0.588 ± 0.02	0.608 ± 0.01	0.609 ± 0.01	0.610 ± 0.01

**FIGURE 4:**
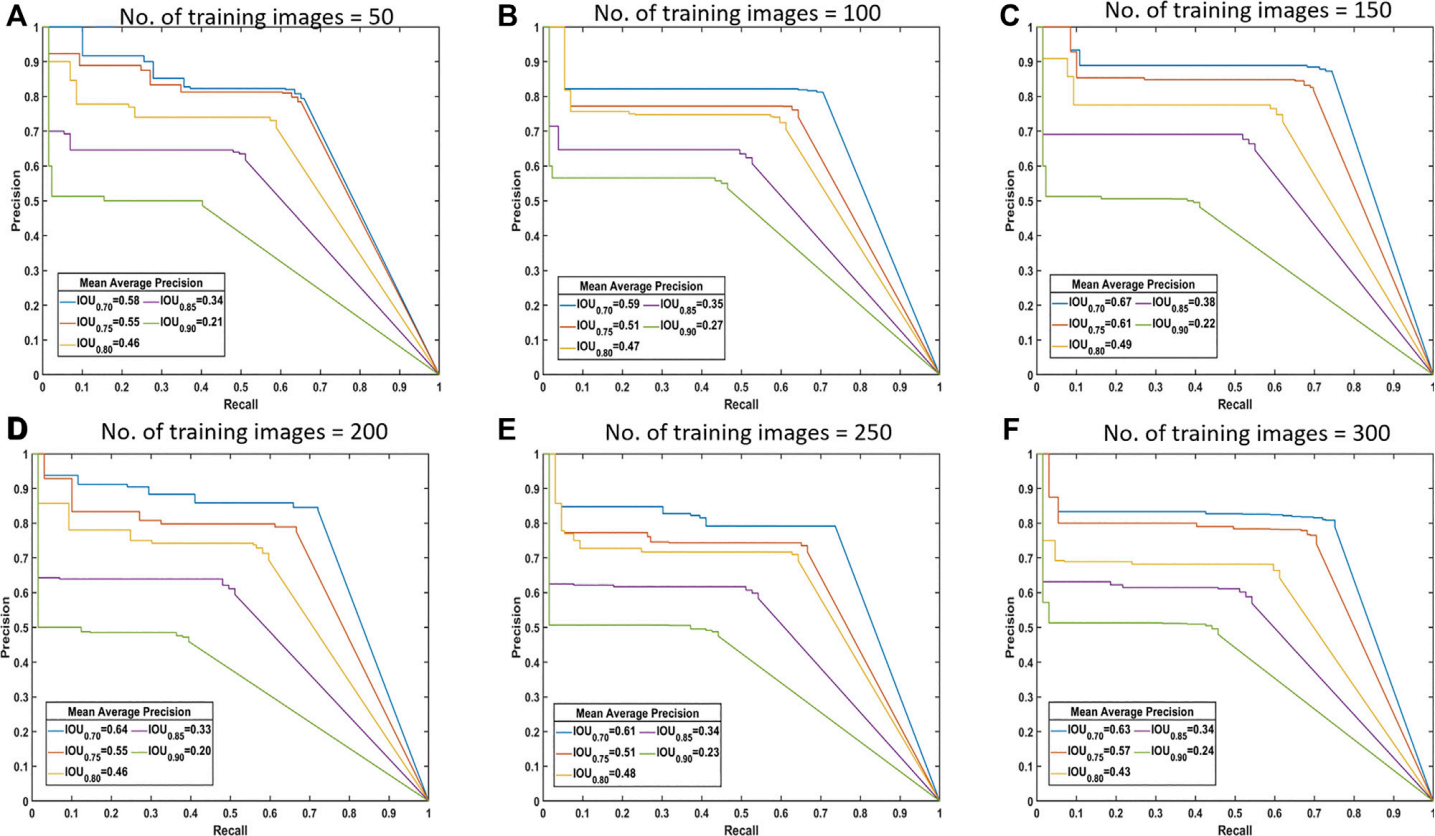
Precision-recall (PR) curves. These are standard PR curves with IoU thresholds and corresponding AP values. As we increase the threshold, the area under the curve decreases, and after a certain number of training images, there is less variance.

Drawing the PR curves using these models can provide a more informative comparison of the segmentation performance of the models. Figure 4 shows the PR curve at higher IoU threshold values than the one used to obtain the mean IoU for both the classes shown in the above table. This was specifically done to observe the model performance in case a high IoU thresholds are considered at the time of segmentation. A model with 100 images can be considered as poor compared to a model with 300 training images, but models with 250 and 300 images performs nearly identical to each other in the segmentation task.

**FIGURE 5:**
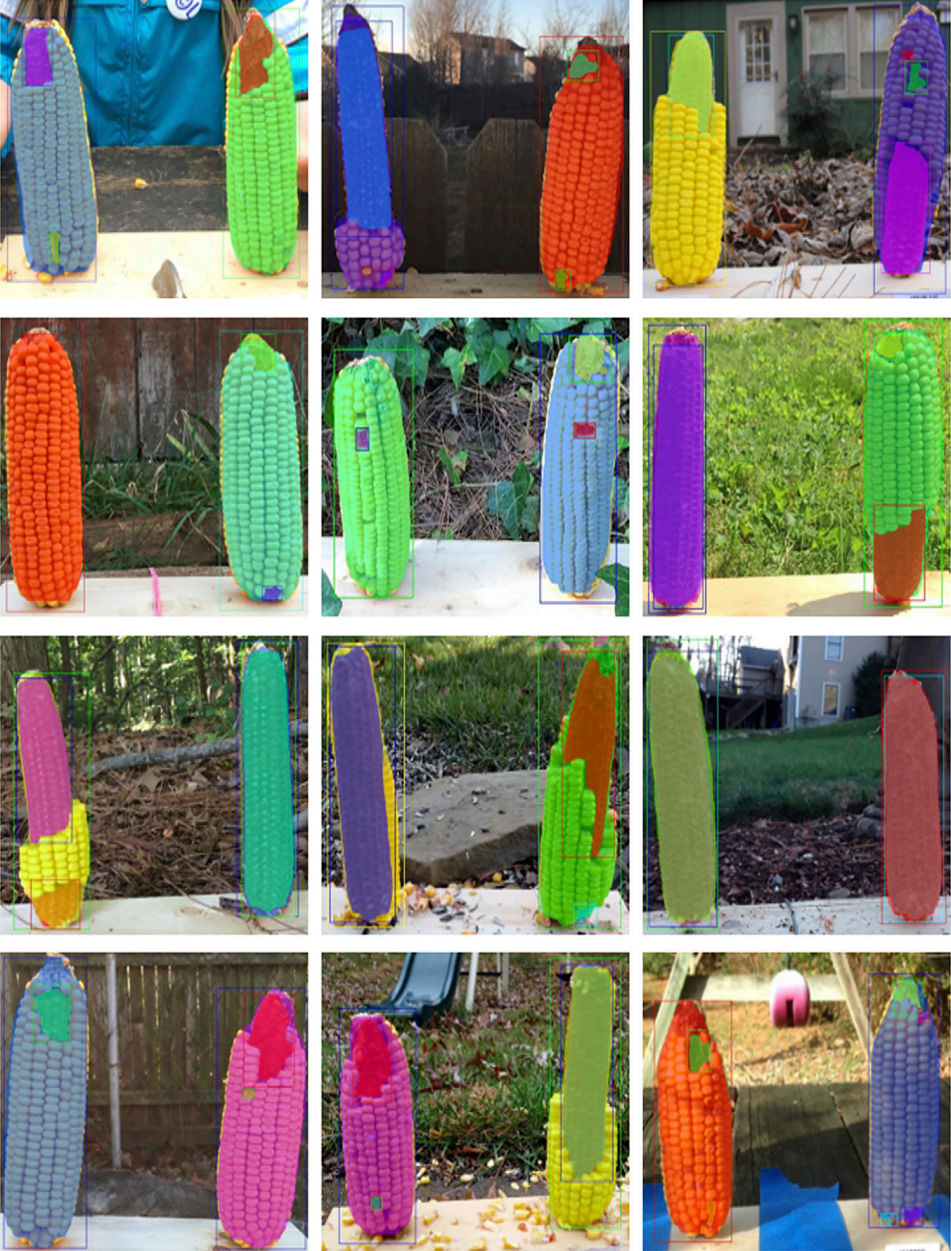
Segmentation results. Representative examples from test data with predicted masks.

From these observations, intuitively it is possible to answer the question of the number of images required for training. For segmentation of the dataset, all of the considered metrics showed that there was little difference in performance from 250 images to 300 images. There is no standard (fixed) ratio for selecting this number in most of the domains and thus we find the sufficient training size by performing these experiments. Hence, it can be said that, about 200–300 images are required for adequate segmentation in this case. Figure 5 shows some of the segmentation outputs from the test dataset. It can be seen that this model identified the bare parts very accurately, which is the key for calculating the consumption of corn ears.

### 3.3 Corn Consumption Estimation

**FIGURE 6:**
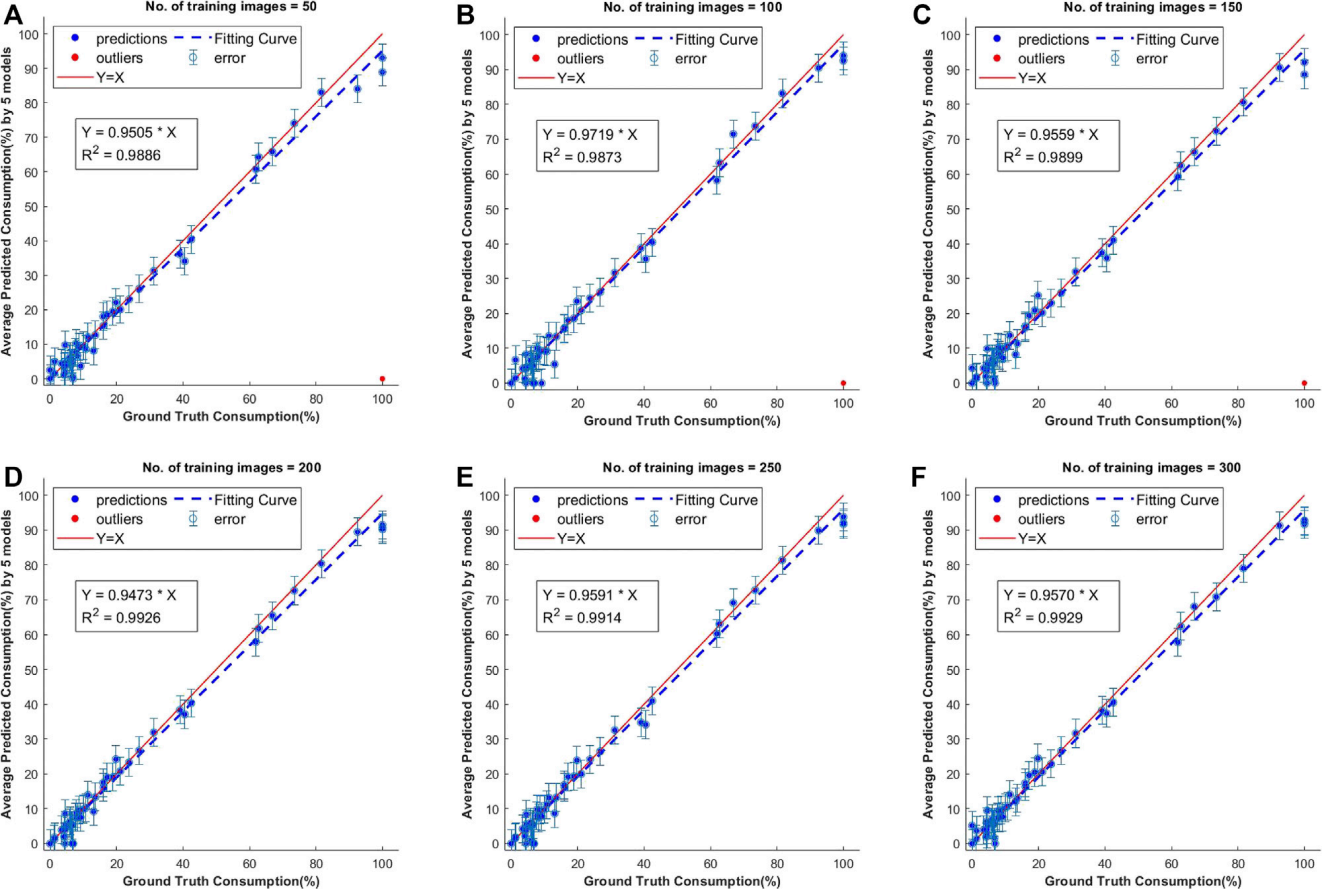
Scatter plots of predicted vs. ground truth values of consumption ratio using models with different number of training images. The outliers shown in red are estimations when the model has failed to predict the class instance mask and they were not considered for computing the *R* ^2^ values.

The consumption values of 50 individual corn ears were obtained from the segmentation results of 25 test images and comparison was done with the average of human labeled ground truth values. The test images were annotated with Approach 1 and the consumption was calculated using these manually labeled masks. The results of this comparison showed that all of these models performed well on the test dataset but the models trained with lower number of images had a few outliers (Figure 6). The best *R* ^2^ value of 0.9929 was achieved with models trained on 300 samples. There was not much difference in consumption calculations with these models, but it can be seen that there were more instances of failure in segmentation for the models trained with fewer images.

#### 3.3.1 Outlier Analysis

**FIGURE 7:**
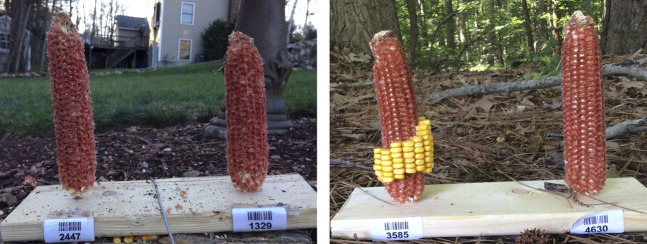
Failed segmentation examples. The right ears in both images were completely consumed, but the models failed to identify it as both whole corn and bare cob part.

When the training set was smaller in size, there is a missed segmentation of bare cob with *Approach 1*. Figure 7 shows the two images in which the right corn ear was not identified as *Whole corn* and *Bare cob* (which are the two classes we have considered). As per Approach 1, these corn ears belong to both the categories but the models with fewer training samples could identify that corn ear in only one of the classes, i.e., whole corn, because of which the predicted consumption is zero. As the segmentation itself failed, this is an outlier in consumption estimation and thus omitted from the scatter plots discussed above.

It can be seen that the right side corn ear in both images has a similar shaped structure in the background—a tree stem attached directly to the corn ear. This could be one of the reasons it is ambiguous at the pixel level. Also, these test images belong to 95–100% consumed category, for which we did not have abundant training data. In the total of 300 training images, we only had 10% of such images, and may be fewer than this when we randomly select a smaller sample size (50–150). This imbalanced training data can be fixed by adding more of such training samples, as shown in the model trained with 300 images, where we did not observe outliers.

#### 3.3.2 Left-Right Comparison

**FIGURE 8:**
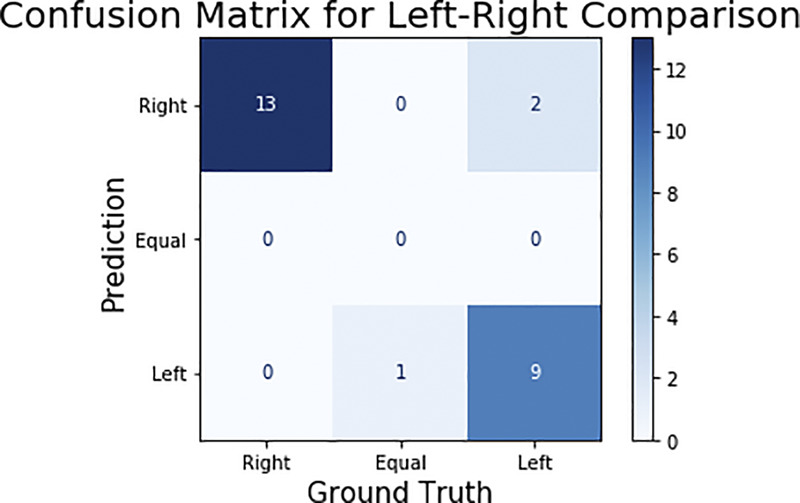
Confusion matrix for left-right corn ear consumption comparison. In ground truth, there were 13 images in which the right ear is eaten more, one image in which both are equally consumed (as shown in Figures 7 and 11) images in which left ear is consumed more.

The GMO and Non-GMO ears were randomly placed by participants on each feeding stand, and the identity of both corn ears in each image was blinded. To perform an unbiased comparison between ears, the model’s output was compared for the consumption of left and right corn ears within each image. The 25 test images were then classified in three categories—right, left, and equal—representing on which side the corn consumed more. The results can be seen in the confusion matrix—among the 25 test images, 22 classified images matched with the ground truth (Figure 8). It should be noted that the remaining three images were misclassified by marginal differences between ground truth and predicted consumption estimation. For example, the image with equal consumption had both the corn ears 100% eaten, while the predicted consumption was 99% and 98% for the right and left ear respectively.

### 3.4 Segmentation of Ambiguous Data

As stated in section 2.1.1, the images that fits certain criteria were used to prepare the models discussed so far. Furthermore, the images in this actual case can have various abnormalities, such as varying light exposure, ambiguous corn ears, varying backgrounds, among others. To verify the performance of this model, a manually generated set of 20 images having corn ears in varying conditions was used and performed consumption estimations of these images. When the consumption of the same set of corn was computed from images taken from different perspectives, it was observed that there was a greater difference between the predicted consumption value and the ground truth. The *R* ^2^ value achieved was 0.88 for this dataset with more than 10 outliers.

**FIGURE 9:**
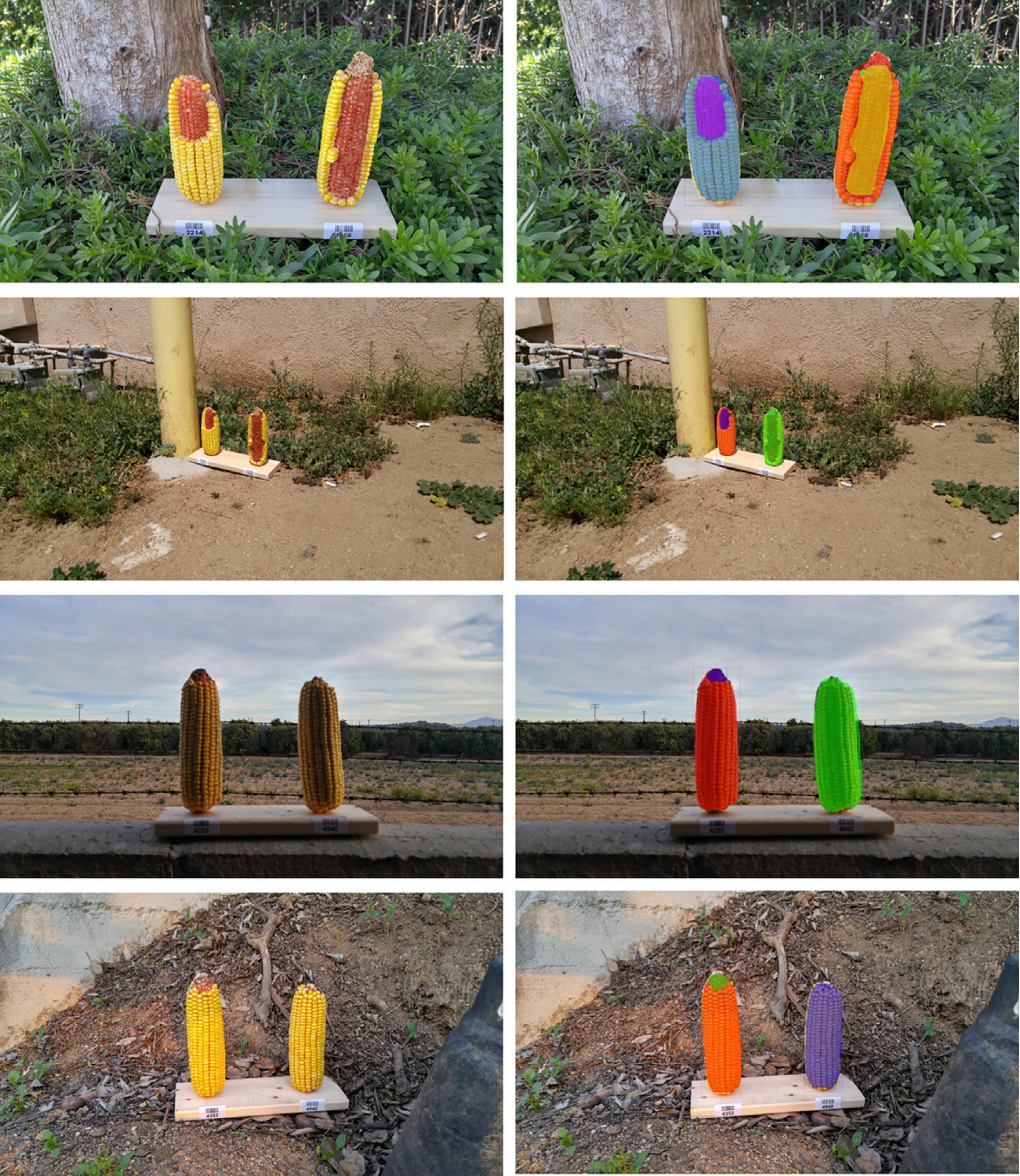
Effect of background and resolution change on segmentation. Left column shows the original image while right shows the corresponding masks. Top two rows are the same set of ears and bottom two rows are the same, but with different background and resolutions.

Representative segmentation results illustrate that the effect of change in perspective as well as effect of brightness on segmentation (Figure 9). The top two images are from the same corn ear set, but in the second row, we can see that the model failed to segment the right side corn due to its low resolution of the actual corn ear (the corn ears only take a very small portion of the whole image). On the other hand, the bottom two rows shows that if an image is taken from an appropriate distance, the model performs well in different conditions of light. Though the training data do not contain such images, the model can still correctly segment these instances and the performance of the models can be improved further by adding more of such images in training.

### 3.5 Discussion

Since the inception of this project in 2018, a variety of new instance segmentation frameworks have been proposed as surveyed by Hafiz and Bhat (2020). For example, Mask Scoring RCNN that also makes segmentation based on detection of region proposals extends further with the inclusion of mask overlap scores and has slightly surpassed Mask R-CNN to achieve 39.6% AP on the same datasets (Huang et al., 2019). Another region proposal-based Path Aggregation Network–PANet achieved top performance in segmentation that enhances feature hierarchy by path augmentation (Liu et al., 2018). Although the above mentioned frameworks perform better in terms of accuracy, the speed of detection remains an issue when the real time segmentation is to be performed. YOLACT (Bolya et al., 2019a) and YOLACT++ (Bolya et al., 2019b) addresses the segmentation speed at the cost of a reduction in AP by prototyping the masks and producing the instance masks with previously predicted mask coefficients. Most recent methods SOLO (Wang et al., 2020a) and SOLOv2 (Wang et al., 2020b) that addresses both speed and AP provides a simple, fast yet strong segmentation framework. This framework follows a rather unconventional approach to assign each pixel a “instance-category” to modify segmentation into a classification-solvable problem. To explore other segmentation approaches that are more recent and are lighter than Mask R-CNN, we performed experiments on available datasets using SoloV2 (Supplementary Table S4). It was observed that SoloV2 segments the Whole Corn class with good precision but does not perform well for the Bare cob class. The result suggests that Mask R-CNN still remains to be a robust instance segmentation method and it performed satisfactorily with a small amount of data. In future studies, however, other newer instance segmentation models could be further explored to improve the segmentation performance.

In this study, the proposed instance image segmentation approach measured how much corn was removed from an ear by comparing bare ear to kernels. The same method could be used to measure yield losses where ears of corn are damaged by hail or partially eaten by pests and wild animals. If further developed by providing appropriate training data, the trained deep learning model could learn to identify particular types of damage. In participatory breeding, some field trials are conducted at remote locations and there is the challenge of measuring phenotypes that would otherwise be easy to measure with people and equipment available at one’s home institution (Ceccarelli, 2015). The method developed in this study would allow farmers and others who are monitoring remote locations to be able to collect images that could be turned into useful data after the images are analyzed by similar deep learning models presented in this study with domain adaptation. A tool could be developed from this study that would allow farmers to take photos of damaged ears and quantify how much yield loss was caused by pests and disease during ear development (Steinke et al., 2017). It should be noted that plant breeding is already incorporating machine learning approaches to analyze and predict phenotypes (Singh et al., 2016; Jiang and Li, 2020), but the proposed approach is unique because it can utilize field-collected image data with varying angles, orientations, and lighting with non-standardized resolutions and uncontrolled background. Therefore, this approach can be useful for decentralized and participatory crop research and breeding.

## 4 Conclusions

In this work, a deep learning based framework to quantify the consumption of corn with a relatively small number of images collected by community scientists was presented and evaluated. The Mask R-CNN model was demonstrated to produce high quality results of pixel-wise segmentation for the challenging task of multi-label segmentation of consumed corn. The two approaches for labeling the ground truth were presented, and it was found that segmenting only the whole corn and its consumed part is sufficient for estimating consumption. The best results were obtained when the training data were sufficient and labeled with high accuracy. The effects of varying light conditions and background were examined and it was found that the Mask R-CNN model, which was not trained with such images, was able to identify certain segmentation instances accurately, and can be improved upon by including such images for further training. The framework developed in this study can be used to predict more samples collected in the GMO Corn Experiment and will produce reliable results more efficiently than manual labeling. Future work will be directed at improving the variation in accuracy as well as testing the visually challenging images, and toward applying the methods developed here along with additional lines of evidence to test the hypotheses that are the focus of the GMO Corn Experiment.

## Data Availability

The original contributions presented in the study are included in the article/Supplementary Material and, further inquiries can be directed to the corresponding author.
