# Supplementary material for: Instance Segmentation to Estimate Consumption of Corn Ears by Wild Animals for GMO Preference Tests
Source: Front Artif Intell. 2021 Jan 29;3:593622. doi: 10.3389/frai.2020.593622 (PMC7941411; doi:10.3389/frai.2020.593622)
Supplement: Supplementary file 1 [file datasheet1.pdf]

Supplementary Material

1 SUPPLEMENTARY DATA

All the annotated images, labels, and source code are archieved in the GitHub repository (`https://github.com/UGA-BSAIL/Corn-Segmentation`) along with the instruction to run pretrained models and train new models for consumption estimation.

2 SUPPLEMENTARY TABLES AND FIGURES

2.1 Statistical analysis of the model performance with various sample sizes

With two-sided t-tests (Table S1-S3), the performance metrics were statistically analyzed to find the significant improvements corresponding to sample size increase.

Table S1. Statistical Analysis using t-test on overall segmentation mAP with various sample size (t-value, P-value)

Sample size	50	100	150	200	250	300
**50**	0,1	**1.93,0.09**	1.12,0.29	3.46,0.009	2.38,0.04	4.3,0.002
**100**	-	0,1	**0.34,0.7**	2,0.08	0.53,0.60	2.9,0.02
**150**	-	-	0,1	**1.7,0.12**	0.7,0.5	2,0.07
**200**	-	-	-	0,1	**1.54,0.1**	0.18,0.8
**250**	-	-	-	-	0,1	**2.34,0.04**
**300**	-	-	-	-	-	0,1

Table S2. Statistical Analysis using t-test on segmentation IoU for Whole corn with various sample size (t-value, P-value)

Sample size	50	100	150	200	250	300
**50**	0,1	**0.97,0.35**	2.36,0.04	2.55,0.03	2.94,0.01	3.71,0.005
**100**	-	0,1	1.46,0.18	1.7,0.12	2.12,0.06	3.02,0.01
**150**	-	-	0,1	**0.35,0.73**	0.85,0.4	1.95,0.08
**200**	-	-	-	0,1	**0.45,0.6**	1.44,0.18
**250**	-	-	-	-	0,1	**1,0.34**
**300**	-	-	-	-	-	0,1

Table S3. Statistical Analysis using t-test on segmentation IoU for Bare cob with various sample size (t-value, P-value)

Sample size	50	100	150	200	250	300
**50**	0,1	**1.93,0.09**	1.12,0.29	3.46,0.009	2.38,0.04	4.3,0.002
**100**	-	0,1	**0.34,0.7**	2,0.08	0.53,0.60	2.9,0.02
**150**	-	-	0,1	**1.7,0.12**	0.7,0.5	2,0.07
**200**	-	-	-	0,1	**1.54,0.1**	0.18,0.8
**250**	-	-	-	-	0,1	**2.34,0.04**
**300**	-	-	-	-	-	0,1

2.2 Performance comparision between Mask R-CNN and SoloV2

To explore other segmentation approaches that are more recent and are lighter than Mask R-CNN, we performed experiments on available dataset using SoloV2. To train a SoloV2 model, we had to convert our data (images and annotation masks) into COCO labelling format. For the sake of simplicity and in the interest of time, instead of converting the entire dataset, we converted a smaller sample size (up to 150).

We compared the performance of the two methods with regard to the mean average precision, and IoU for Whole Corn and Bare Cob classes.

Table S4. Effect of training sample size on segmentation performance of Mask R-CNN and SoloV2 model

**Metric**	**mAP**		**Whole Corn IoU**		**Bare cob IoU**	
**Method**	**Mask R-CNN**	**SoloV2**	**Mask R-CNN**	**SoloV2**	**Mask R-CNN**	**SoloV2**
**50**	0.574	0.09	0.868	0.5792	0.606	0.435
**100**	0.592	0.24	0.876	0.7412	0.636	0.436
**150**	0.588	0.42	0.885	0.815	0.626	0.561
